# A model to explain the challenges of emergency medical technicians’ decision making process in emergency situations: a grounded theory

**DOI:** 10.5249/jivr.v14i1.1604

**Published:** 2022-01

**Authors:** Meysam Safi-Keykaleh, Davoud Khorasani-Zavareh, Zohreh Ghomian, Katarina Bohm

**Affiliations:** ^*a*^ Nahavand School of Allied Medical Sciences, Hamadan University of Medical Sciences, Hamadan, Iran.; ^*b*^ Workplace Health Promotion Research Center, Department of Health in Emergencies and Disasters, School of Public Health and Safety, Shahid Beheshti University of Medical Sciences, Tehran, Iran.; ^*c*^ Department of Health in Emergencies and Disasters, School of Public Health and Safety, Shahid Beheshti University of Medical Sciences, Tehran, Iran.; ^*d*^ Department of Clinical Sciences and Education, Karolinska Institute, Stockholm, Sweden.

**Keywords:** Emergency medical services, Emergency medical technician, Decision making, Pre-hospital, Iran

## Abstract

**Background:**

To manage life-threatening conditions and reduce morbidity and mortality, pre-hospital’s on-scene decision making is an influential factor. Since pre-hospital’s decision making is a challenging process, it is necessary to be identified this process. This study was conducted to explore the model of Iranian emergency medical technicians’ decision making in emergency situations.

**Methods:**

This study was applied through grounded theory method using direct field observations and semi-structured interviews. Purposeful sampling with 26 participants including 17 emergency medical technicians including dispatchers, physicians of medical directions, managers and 1 representative for court affairs was performed. Interviews were lasted from October 2018 to July 2019. Corbin and Strauss approach, 2015 (open, axial and selective coding) were used to analyze data.

**Results:**

A paradigm model was developed to explain the relationships among the main categories. Decision making in the context of fear and concern was emerged as the core category. Unclear duties, insufficient authorities and competencies as well as lack of enough decision making’s protocols and guidelines were categorized as casual conditions. Other important categories linked to the core category were interactions, feelings and “customer focus approach”. Action–interaction strategies were taken by Emergency Medical technicians lead to some negative consequences that can threaten clinical outcome and patient safety.

**Conclusions:**

Based on the finding of this study, Emergency Medical technicians’ decision making in the context of fear and concern, as the core concept of this model, lead to decrease in quality of the pre-hospital services, stakeholders’ dissatisfaction, hospital emergency units’ overload, decrease in reputation of the Emergency Medical Technicians, threat to patient clinical outcome and patient safety. To prevent of these negative consequences, facilitation of the Emergency Medical Technicians’ on-scene decision making is recommended.

## Introduction

Emergency Medical Services (EMS) centers were established to provide on-time and rapid services to the patients and injured from the scene to the hospital.^1^ These centers can reduce significantly morbidity and mortality with providing pre-hospital cares in life-threatening conditions.^2^ To manage life-threatening conditions, save lives and reduce morbidity and mortality, rapid and accurate on-scene decision making is an important and influential factor.^3^

Decisions about the type and priority of emergency medical interventions require triage.^4^ As the conditions of some patients are complex and variable, the technicians' decision changes frequently, which can lead to technician error.^5,6^ Decision making errors threaten patient’s safety and Emergency Medical Technicians (EMTs)’ reputation.^7-10^ However, proper emergency medical decision-making leads to prevention of hospital emergency unit overcrowding, increased quality of health services and ultimately reduced morbidity and mortality.^9,11^

EMTs' on-scene decision making is influenced by incredible barriers and challenges.^7,12,13^ These factors influence the thinking, feeling and performance of the EMTs and so, increase their decision error, especially in some interventions including endotracheal intubation, mediation and no transportation.^14,15^ Facing these barriers and challenges lead to the EMTs’ concern and fear which of these fears have a profound impact on patient outcomes.^16,17^ As stress and anxiety often arise at the time of providing on-scene health services,^18^ identifying these factors are necessary.

There is a paucity of studies in the field of EMTs’ on-scene decision making that have provided a good overview of the factors affecting dispatch services and transmission, but the decision-making process at the scene was not explored yet.^19-21^ While EMTs’ decision-making is a dynamic and on- going process starting from the scene until the patient is delivered to the hospital.^20,22^ This process will be influenced by multiple factors, so will have multiple outcomes. Understanding this process is crucial and can significantly address challenges and improve patient outcomes. Therefore, this study was conducted to design EMTs' on-scene decision making model in order to simplify the relationship between the components of this process.

## Methods 

**Study setting**

In Iran, national EMS system provides free services for patients and injured people from scene to hospital. In the provinces and cities, EMS centers are under supervision of universities medical sciences and national EMS system. EMTs including diploma(basic), nurses with associated degrees(intermediate), bachelor’s and master’s degrees (advanced/ paramedic) provide services in dispatch centers or on scene. There is a dispatch center in each provincial center or cities which response to calls, provide counselling, dispatch EMTs and coordinate dispatched ambulances.

Two EMTs in each ambulance were dispatched to the scene who should get advice from general physician as Medical Direction’s Physicians (MDPs) of Dispatch in the provinces’ EMS center. In the cities’ EMS centers. EMTs typically operate independently due to lack of MDPs and communicating challenges. General physicians did not attend the ambulance and most of them as a medical director provide online radio or telephone counselling to EMTs at proveniences’ EMS centers and some of them are as head of emergency centers. There is no general physician in the cities’ EMS center.

**Study design**

A grounded theory method based on Corbin and Strauss approach in 2015 was conducted for data gathering. This method is useful for achieving new area or exploring new perspectives of known field.^23,24^

**Participants’ selection**

Maximum variety sampling method was performed, of which 26 participants including Emergency Medical Technicians, Medical Direction’s Physicians (MDPs), dispatchers, representative for court affairs and EMS’ managers were chosen according to purposeful sampling (Table 1). Having practical or theoretical experience, being verbal and participations’ willingness were inclusion criteria. Moreover, observational field note by means of triangulation was used for data collection as well as data validation. Saturation principle was used for concept saturation, of which data saturation was reached after 26 interviews and 10 filed observations.

**Table 1:** Demographic participants’ characteristics on the study of model of emergency medical technicians’ decision making in pre-hospital emergencies.

Participants characteristics	Number (Percent)
**Position**	
Emergency medical technicians	17 (63.38%)
Medical Direction’s Physicians (MDPs)	2 (7.69%)
Dispatchers	3 (11.53%)
EMS’ managers	3 (11.53%)
Representative for Court Affairs	1 (3.84%)
**Sex**	
Male	21 (80.77%)
Female	5 (19.23%)
**Age**	
25-35	13 (50%)
36-45	10 (38.46%)
46-55	3 (11.54%)
**Work experience (Years)**	
1-10	7 (26.92%)
11-20	15 (57.70%)
21-30	4 (15.38%)

**Data collection**

Data were collected through in-depth interviews and field observations. Interviews were done in Farsi at the participants’ workplaces. Initially three unstructured interviews were conducted. These first three interviews helped to identify interview guideline and important concepts that effect on-scene decision makings. Following that 26 semi-structured interviews were conducted using interview guideline. Interviews were started with general question about participants’ experiences of on-scene decision making process. Following that, to explore participants’ experiences probing questions were performed. Examples of questions were including “What are your challenges at the time of on-scene decision making?”, “What factors effect on your on-scene decision making in emergency situations?”, “What strategies do you use to deal with on-scene decision making’s challenges?” The interviews’ duration lasted between 45 and 75 minutes from October 2018 to July 2019. Each recorded interview was listened several times and transcribed verbatim by the principal investigator (PI). The PI started observation to take field notes after three interviews. Moreover, in order to saturate the concept by means of triangulation, ten observations also conducted from November 2018 to March 2019. In order to do that, PI took part in the EMS’ mission as the participant as observer. In this regard, all relations and interactions were taken as the field notes.

**Data analysis**

Data analysis was carried out simultaneously and immediately after data collection. The PI listened the audio files several times and compared transcribed interviews with recorded digital audios file. Based on Corbin and Strauss recommendations in 2015,^23^ continuously during data analysis. Regarding open coding, transcribed interviews and filed note observations were analyzed line by line. In this regard, MSK and DKZ discussed interviews’ process and codes extracted to provide the guide of future interviews and analysis. Both are experts in the field of health in emergencies and disasters. Translate and back-translate was also carried out by KB who is expert in the field of emergency medicine and English language. As a result of the researchers’ engagement and their consensus, the extracted codes were integrated in sub-categories and categories. Following that, via axial coding, the sub-categories and categories were compared and categorized based on their similarities and differences. Finally, based on selective coding, the link of categories and core categories were obtained and after that conceptual model was designed based on initial theoretical structure. Following that the paradigm model was presented to visualize the core category that is the central and main phenomenon of study.^25^ This model presents the decision making in the context of fear and concern. This core category is affected by Causal condition as a group of situations that influence decision making in the context of fear and concern. Context conditions as a component of the model, refer to a group of conditions that the phenomenon is raised and people respond to it via some strategies. Intervening conditions are facilitators or barriers action-interaction strategies which are purposeful acts that are taken by people to resolve a problem and lead to a number of consequences.^25^

**Trustworthiness**

Four strategies including credibility, confirmability, dependability and transferability were applied to achieve trustworthiness.^26^ To achieve credibility, data collection triangulations including interviews and observational field, researchers’ eligibility in the field of health in emergencies and disasters as well as their prolonged engagement with data was employed. In addition, member checking, peer checking and finally expert checking were applied to improve credibility of the findings. To determine the data consistency, beside the PI other team members, as external auditors, checked generated codes, categories and sub-categories. Expert check was carried out by the opinions of research supervisor, DKZ and KB on the findings. To establish Confirmability, triangulation for data collection including face-to-face interview, bracketing principle and peer evaluation were used. Transferability was achieved using detailed explanations of the data collection, analysis and result interpretation. Finally, dependability was met thought triangulation, code-recode strategy, and peer evaluation.

## Results

This study explored the EMTS 'on-scene decision making process. In the initial analysis, 1352 codes were extracted and finally classified into six categories and 21 sub-categories. “Decision making in the context of fear and concern” was defined as a core category. This type of decision means that most EMTs are concerned about the consequences of their decisions due to ambiguous tasks and responsibilities, insufficient authority, competency and decision-making protocols. These conditions are in the setting concluding lack of trust and inadequate supportive rules for EMTs. Intervening conditions including interactions, fillings, and “Customer focus approach” intervene in the decision making. The participant number were included (by P) for each quote and (FNO) for each field notes observation.

**Figure 1:**
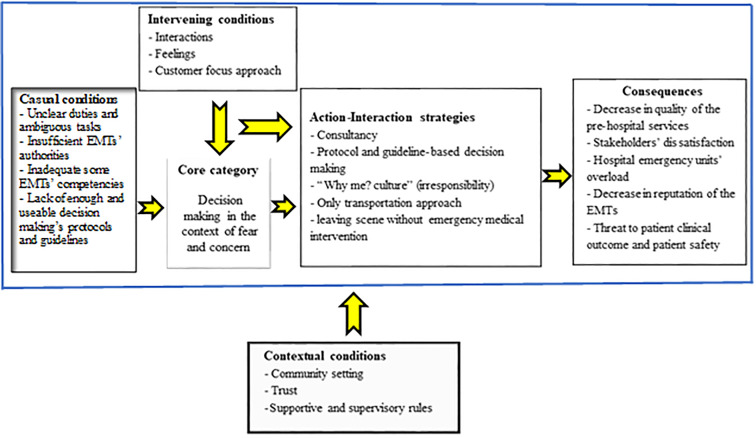
The paradigm model of EMTs’ on-scene decision making process in emergency situation in Iran

Based on the findings of this study, in dealing with fear and concern, EMTs adopt two types of action-interaction strategies in order to manage their stress and anxiety. They may first consult with their co-worker, MDP, or dispatch operator. If this strategy does not solve the problem, they will refer to the existing protocols. Due to the findings of this study, there are many weaknesses and barriers to these strategies, so due to frustration, some EMTs may take the strategies including irresponsibility, only transportation approach and leave the scene and transfer the patients without medical intervention. All of these strategies lead to negative consequences include reduced quality of EMS services, reduced stakeholders' satisfaction, hospital emergency unit overload, reduced EMT reputation and threat to patient safety and their outcomes (Figure 1).

**Causal condition**

In this study, factors including unclear duties and ambiguous tasks, insufficient EMTs’ authorities, inadequate some EMTs’ competencies, and lack of appropriate on-scene decision-making protocols and guidelines lead to decision making in the context of fear and concern. According to EMTs, unclear duties and responsibilities are one of the most important factors that cause fear and concern at the time of decisions making. The EMTs are not completely aware of their duties and often their intervention is not legal. They described that multiple and sometimes indefinite responsibilities not only lead to neglect of necessary interventions, but also lead to excessive stress and anxiety.

*"…I don't know exactly what is my duty and responsibility. If occurred complication in a patient due to my intervention and its possible failure, would we be prosecuted and compensated? ... What duty do we have for patients who refuse to be cared? Whether a patient's endotracheal intubation or medication is illegal without a MDP’s advice…? ". (P3)*

Inadequate authority in contrast with delegated responsibility, inadequate competencies including knowledge, skills, experience and accurate judgment were identified as four important factors influencing the decision-making process of EMTs. The EMTs were concerned about the lack of sufficient authority. They believed that delegating multiple responsibilities without giving them sufficient authority would deprive many patients of emergency medical interventions and cares. According to participants and observation field notes, some EMTs were insufficiently competent to provide emergency interventions and cares. They declared that clinical judgment, experience, knowledge and skills of effective communication and scene management ability are critical to making the right decision in a timely manner. Field observations showed that there were cases of poor emergency scene management when assisting the patients that their family were agitated, which resulted in violence against EMTs.

*"Some co-workers have enough experience and great skill to manage the scene. Judging right in spite of the patient's anger and illogical laypeople’s interference is of important. ...sometimes a patient does not have need an intervention and special care, but unfortunately, we don't have enough authority to leave such a patient. Sometimes the patient doesn't have to be taken to the hospital, but because we don't have the enough authorization, we are afraid of not taking the patient to the hospital." (FNO2)*

According to the EMTs, sometimes making a decision to manage the patients is very difficult and EMT's consultation may be ineffective, so guidelines and protocols are very helpful. If decisions are made on the basis of scientific protocols and guidelines, in addition to appropriate medical interventions, concerns and fears of EMTs about the negative consequences of their interventions will be eliminated. In other words, EMTs can make better decisions with these supporting documents. The lack of protocols for some emergencies and deficiencies in existing protocols, was a major challenge of decision making.

*"... sometimes I talk to my colleague about the patient, but we really don't know what to do ... a guidance can be very helpful, but the number of our pre-hospital emergency guidelines and protocols are limited ... the available guidelines and protocols because of their simplicity or complexity are unusable." (p16)*

**Contextual conditions**

In this paradigm model, community setting, trust, supportive and supervisory rules were identified as contextual conditions. The EMTs were extremely unsatisfied with the irrational and sometimes violent intervention of laypeople on the scene. Distress caused by irrational interferences disrupts emergency medical cares and results in rapid transmission without medical care. Another important aspect of community setting is the level of public education and awareness. The participants believed that community perception and its’ attitudes toward the importance of pre-hospital cares have an important role in their cooperation and collaboration. In the field observation, the involvement of laypeople with severe verbal violence were observed.

*"Unfortunately, sometimes people angrily disrespect us. Although we do our best to help a patient with life-threatening conditions, they prevent us by irrational interferences. I am sure that the main reason for these behaviors and interferences is the low understanding and awareness. “(P10)*

The findings of the study showed that trust had a significant impact on EMT's decision making. All participants believed that lack of trust is a major barrier to participatory decision making. They mentioned that there is no sufficient trust between EMTs, dispatchers, MDPs, and hospital emergency physicians. Some MDPs do not trust the technician's description and only recommend transferring instead of providing medication and medical advice. Lack of proper decision-making condition due to insufficient trust leads to fear and anxiety of EMTs. The EMTs believed that there is no a " Chain of trust " to facilitate the decision-making process, so people do not have trust to knowledge, skills and importance of EMTs’ medical approaches and cares.

*"I believe that team-working in the pre-hospital emergency services is not possible without trust. I think there is no sufficient trust among our staff. When a physician does not have enough trust to the technician's report, how to order medical intervention. (P9) ... People do not trust our EMTs' capability ... because of the weak trust chain, sometimes some patients and their companions are not honest... ". (P6)*

This study addressed that supervisory and supportive laws play an important role in emergency medical decision making. Lack of clear rules and regulations to determine the scopes of EMTs’ duties and lack of proper professional liability coverage by insurance companies were obtained as one of the most important factors leading to EMTs’ concern and fear. The EMTs stated that they were constantly afraid of the possible negative consequences of their decisions and interventions. Uncertainties at the time of dealing with an alone elderly or child patient who refuse to receive care, lack of clear rules related to end-stage patient and cardiopulmonary arrest who had clinical death were the most important stressors for decision making.

*".... We don't know what the legal consequences of leaving the alone patient who refuse to receive care or transport ...” (P1) …there are many missions that the patient is dead and there is nothing to do, but we transfer them to hospitals due to lack of supportive laws and insurance." (P21)*

**Intervening conditions**

Interactions, feelings, and the “Customer focus approach” are three important intervening factors in EMTs’ on-scene decision-making that lead to Action-Interaction strategies. According to participants, although some patients don’t not have emergency medical problems, they or families tend to exaggerate their problem to use free services. On the other hand, some people behave violently with the EMTs that these verbal and non-verbal violence were identified as the most important disrupting interactions factor and consequently making decisions with fear.

*"Sometimes some patients pretend to be ill that we find out thorough physical and psychological examination...” (P13) Sometimes we face violent and inappropriate behavior from laypeople and bystanders that lead to stress, fear and wrong decisions." (P4)*

The EMTs declared that their decisions and actions are also influenced by their feelings. The findings of this study indicated that feelings such as altruism and empathy play an important role in the decisions of EMTs. Seeing patients' suffering affects the feelings and focus of the EMTs. Another negative feeling reported by EMTs was that they felt unhelpful and sense of inadequacy. They were sometimes unable to do something, in spite of being aware of patient's needs. 

*"... Pain and groaning of some patients, especially those who are end-stage and those who are really poor affect me. We put all our focus and efforts on patients. Nevertheless, when I feel like some managers don't care, I'll be disappointed... “(P16)*

Excessive attention to the satisfaction of patients and their families by EMS system managers and policymakers, was noted repeatedly by the EMTs as "Customer focus approach”. Most participants claimed that some EMS Managers and health policy makers only want to obtain the satisfaction of caregivers. The data extracted from observations and interviews showed that some managers neglected the EMTs’ satisfaction that had negative effect on EMTs’ motivation and so their decisions. 

*"... There are many cases with no emergencies that request an ambulance several times a week. Some patient or families threaten us very badly ...” (P14) … in the face violence, the executives say, in any case, they are sick and your duty is providing services ...” (P20)*

**Action-Interaction strategies**

Based on the participants and fieldnotes, action strategies including protocol and guideline-based decision making, consultancy, and interaction strategies including “why me culture?”, only transportation approach and leaving scene without emergency medical intervention were obtained. EMTs act based on action strategies to overcome decision-making challenges and barriers. If consulting with another EMT could not resolve decision-making problems, they will ask dispatcher to consult with a physician, but most EMS centers do not have a physician. Protocol and guideline-based decision-making is another action strategy that face challenges such as the difficulty of using paper-based protocols, forgetfulness of protocols, the complexity of some protocols.

*"Sometimes I can't make a decision. I consult with my colleague. Sometimes my colleagues ask me what to do. We sometimes ask dispatcher to consult with a physician, but most of the time it is not possible to consult with...” (P2)*

In the case of EMTs’ frustration to adopt an action strategy, they adopt a strategy of irresponsibility that was coded as "Why me? Culture”. Most participants declared that because of distress caused by bystanders’ irrational interference, they refused to medical emergency interventions and their responsibility. Fear of the decision’s negative consequences, feelings, people's interference and scene conditions are factors that lead to the only transportation approach so that EMTs sometimes leave the scene without care or intervention.

*"In my opinion, the uncertainty, the pressure of the scene, the fear of the negative consequences of the decision have led some of our colleagues to not take responsibility...”. (P11)*

**Consequences**

According to the most participants, the EMTs’ frustration in on-scene decision-making process lead to decrease in quality of the pre-hospital services, stakeholders’ dissatisfaction, hospital emergency units’ overload, decrease in reputation of the EMTs and threat to patient clinical outcome and patient safety. The participants believed that strategies such as "why me? Culture", only transportation approach, and leaving the scene without providing emergency medical cares would lead to decrease in quality of the pre-hospital services. The EMTs’ concern and fear lead to make hasty or incorrect decisions and so, threat to patients’ safety. Rapid and correct on-scene decision-making lead to on time medical intervention and emergency care so it concluded to caregivers’ satisfaction. Furthermore, as a result of correct decisions, on time, enough and adequate pre-hospital cares prevent from transportation of all cases to the hospital and hospital emergency unit burden.

*"A lot of times we have to move patients as soon as we arrived to the scene…” (P10) …transporting all cases to the hospital, cause overcrowding and overloading of the hospital…” (P22) … inadequate pre-hospital cares lead to threat to patients’ safety and patients’ dissatisfaction”. (P10)*

Another negative consequence was the reduction of EMT reputation. In other words, some people do not trust the knowledge and competence of EMTs. The EMTs claimed that some people do not believe in them. Furthermore, attitudes and perceptions of hospital staff including nurses and physicians based on inadequacy of EMTs' competence were unbearable to EMTs. 

*"People think we are just drivers. I think, the main reason of this attitude, is transporting the patients without enough emergency cares”. (P1)*

## Discussion

The findings of this study indicated that some EMTs face fear and concern due to unclear duties and ambiguous tasks, insufficient authority, inadequate competencies, and deficiencies of medical protocols. These factors are in the setting of inadequate trust, inadequate supervision and lack of supportive laws. The findings suggested that intervening factors such as interactions, EMT feelings, and “Customer focus approach” increase EMTs' fear and concern at the time of decision making. Technicians may adopt strategies such as consultancy or protocol referencing to overcome this fear. If these strategies fail, they take the irresponsibility approach and leave the scene without medical intervention. Based on the extracted model, these strategies concluded to decrease in quality of pre-hospital services, reduced stockholders’' satisfaction, hospital emergency unit overload, decrease in EMT’s reputation and threat to patient’s safety and outcomes.

One of the most important factors affecting the fear and concern of EMTs was insufficient authority. The EMTs believed that despite numerous and critical tasks, they did not have sufficient authority to provide medical emergency interventions. sufficient authority, especially in pre-hospital setting where patients are critical and in a life-threatening condition, is essential because it is not possible to delegate vital responsibility and provide lifesaving care without sufficient authority.^27^

Lack of sufficient and usable protocols and guidelines were categorized as the casual conditions. The EMTs have to make decision based on their judgment that is not evidence-based. So, the EMTs fear of legal consequences due to possible threat to patients' safety and dissatisfaction. The studies suggested that protocols and guidelines not only facilitate the physicians’ and nurses’ work but also provide better outcomes for patients and supportive space for health providers.^28-30^ A study that explored the experiences and perceptions of health providers in Iran, showed that decision-making protocols were complement to dispatchers’ experience.^31^ Therefore, 

Based on the experiences of the participants, some EMTs did not have sufficient competency including knowledge, skills, experience and the power of self-management, which were important causes for fear and concern. However, the findings of other studies that explored the competencies of pre-hospital providers, emphasized that having enough competencies is vital to provide on-time and correct clinical judgment.^32,33^ In order to improve EMTs competencies, it needs to continuous educational plan for both knowledge and particularly their skills, which is in line with previous study in pre-hospital phase improvement in Iran.^34^

As a contextual condition, community setting is one of the affecting factors on EMTs' on-scene decision making process. One of the aspects related to community setting is the bystanders and laypeople interferences in relief and rescue operations. The reason for people's interference was lack of their awareness of the EMTs’ duties and importance of pre-hospital cares. Other studies in the field of road traffic injuries in Iran also mentioned that bystanders’ interference has a negative role in pre-hospital emergency services^21,35^ because it led to over/under triage and EMT's confusion.^36,37^

The findings of this study showed that pre-hospital emergency services should be provided in a context of mutual trust. This chain of trust should exist between providers and caregivers as well as intra-organizational EMS staff. The studies claimed that trust is a vital component in the provision of health services and the promotion of stockholders’ collaboration.^38,39^

Based on this study findings, the cause of the malingering was getting rid of or obtaining specific conditions. Because malinger patients are resistant to EMTs’ intervention and cares and their families are believed in them, technicians face serious problems when dealing with these people.^40,41^ Empathy with such a patient and his family as well as respectful communication were recommended.^42,43^

Threats and violence against emergency personnel were identified as an aspect of negative and stressful interactions. The studies showed that fear, anxiety, sleep disorders, decrease in self-esteem are the negative consequences of exposure to violence.^44-46^ According to the findings of this study, most violent technicians were dissatisfied with poor support and follow-up of their organization. However, according to the studies, organizations should strive to satisfy their employees.^47-49^ Based on that, it is recommended for passing supportive law for EMTs in their duties and particularly on-scene protection of EMTs.

The findings of this study revealed that EMTs have adopted various strategies when faced with decision-making barriers that one of the most important was use of protocols, but most technicians stated that the protocols are inadequate and complex. A study was conducted to explore the feedback system between dispatch centers and ambulances in Sweden, also described that some EMS personnel did not use decision-making and dispatching protocols because of their complexity.^50^ A medical protocol should take all the clinical aspects of an emergency situation in order to make the best decision and thus the best possible service.^51^

Consulting with Dispatch Operators and MDPs was another EMTs’ strategy to take guidance and sometimes get rid of the legal consequences of decisions thorough delegating responsibility to others. Responsibility is known as the cornerstone of provision of medical service.^52,53^ However, the findings of this study showed that technicians face the mistrust of some physicians and communication barriers. In line with our findings, similar studies mentioned legal Issues for the medical director.^54,55^

The analysis of the findings of this study indicated that some EMTs adopt an only transportation strategy with minimal and sometimes no care and medical intervention due to irrational interference of laypeople and bystanders. Although technicians provide essential services to patients in the ambulance during transportation, deprivation of critical care on the scene results in reduced quality of service, threat to patient safety. In line with the findings of this study, the studies highlighted that hospital emergency units’ overload, wasting time and facilities to transfer non-emergency patients is one of the negative consequences of this strategy.^15,56-58^

**Study strengthens and limitations**

The qualitative approach was used to explore the model of EMTs’ on-scene decision making in a middle-income setting. To our best knowledge, this study is the first study on EMTs’ on-scene decision making in Iran that was employed both interview and observation. To have maximum variation, the participants were selected among EMTs, dispatchers, managers, MDPs and policymakers. To achieve consistency and the credibility of the data, different methods including constant comparative analysis, member check, and peer review were used. One potential limitation of the current study is that, this study conducted in the Iranian context that can be limited in generalizability to other countries. In qualitative study, the scope is not generalization, however, it seems that given the shared problems, the results can be applicable in low- and middle-income countries.

## Conclusion

This study designed a paradigm model to explain the process and to explore the relationships between different components and affecting factors on EMTs' on-scene decision making process. Technicians' decision making in the context of fear and concern leads to action-interactive strategies that will ultimately lead to stockholders’ dissatisfaction, decrease in EMTs’ reputation, threat to patient safety and related outcome. According to the model, unclear tasks, and insufficient EMTs’ authority, inadequate some EMTs’ competencies, and lack of sufficient protocols and guidelines were categorized as Casual Conditions. Promoting EMTs’ competencies, delegating sufficient authority, and passing supportive rules for emergency personnel are suggested. Based on this model, there are several contextual and intervening conditions that effect on EMTs’ decisions. Finally, to facilitate on-scene decision making process, it is necessary that some strategies were implicated in national EMS system.

**Abbreviations**

EMS: Emergency Medical Services; EMTs: Emergency Medical Technicians; MDPs: Medical Direction’s Physicians; DKZ: Davoud Khorasani-Zavareh; MSK: Meysam Safi-Keykaleh; ZG: Zohreh Ghomian; KB: Katarina Bohm.

**Acknowledgements**

This study is part of the Ph.D. thesis in School of Public Health and Safety. The authors would like to thank Shahid Beheshti University of Medical Sciences as well as all the participants in this study especially the EMTs.
